# Classical biomarkers and non-coding RNAs associated with diagnosis and treatment in gastric cancer

**DOI:** 10.32604/or.2025.063005

**Published:** 2025-04-18

**Authors:** JINGDAN QUAN, ZIXIN WAN, WEI WU, XINYUAN CAO, JIAYUAN QIU, XIAOYE LIU, ZHIWEI ZHANG

**Affiliations:** 1 Key Laboratory of Cancer Cellular and Molecular Pathology in Hunan Province, Cancer Research Institute of Hengyang Medical College, University of South China, Hengyang, 421001, China; 2 Hengyang Medical College, University of South China, Hengyang, 421001, China

**Keywords:** Gastric cancer, Biomarkers, Immune checkpoint molecules, Diagnosis, MicroRNA, CircRNA, LncRNA, piRNA

## Abstract

One of the most prevalent malignant tumors worldwide, stomach cancer still has a high incidence and fatality rate in China, and the number of young people developing early-onset gastric cancer is steadily increasing. The 5-year survival rate of stomach cancer is typically 30%–35%, the prognosis is bad, the patients’ quality of life is low, and the progression of advanced gastric cancer cannot be effectively managed despite the use of surgical surgery, chemotherapy, and other medicines. We urgently need molecular biomarkers with high specificity and sensitivity to increase the early gastric cancer detection rate, extend patient survival, and improve patient quality of life. The initial diagnosis of gastric cancer primarily depends on gastroscopy and biopsy, and invasive procedures cause significant discomfort to patients. Similar to this, treating advanced and metastatic stomach cancer is a pressing issue that requires attention. More and more immune checkpoint molecules have been discovered, and corresponding inhibitors are gradually being applied to clinical diagnosis and treatment. Recently, some non-coding RNAs have begun to be used as new targets for the treatment of gastric cancer. Some non-coding RNAs are highly present in the serum or urine of gastric cancer patients and can be used as diagnostic markers or prognostic indicators. Many clinical trials targeting non-coding RNAs have also shown good therapeutic effects. In general, targeting non-coding RNAs has shown good therapeutic effects. The biomarkers for gastric cancer detection and treatment are reviewed in this article, focusing on the new non-coding RNAs used in diagnosis, prognosis, and treatment. Patients with stomach cancer should have access to more precise and efficient diagnosis and treatment choices as a result of ongoing technological advancements and thorough research.

## Introduction

According to the latest global cancer statistics, stomach cancer has the fifth-highest incidence and fifth-highest mortality rate worldwide. Over 950,000 new cases of stomach cancer and roughly 660,000 deaths occurred globally in 2022. Additionally, there are significant disparities between genders in the incidence and death of gastrointestinal cancer. Incidence and mortality rates for stomach cancer were 1.84 times higher in males than in women, according to global cancer data for 2022 [1].

**Figure 1:**
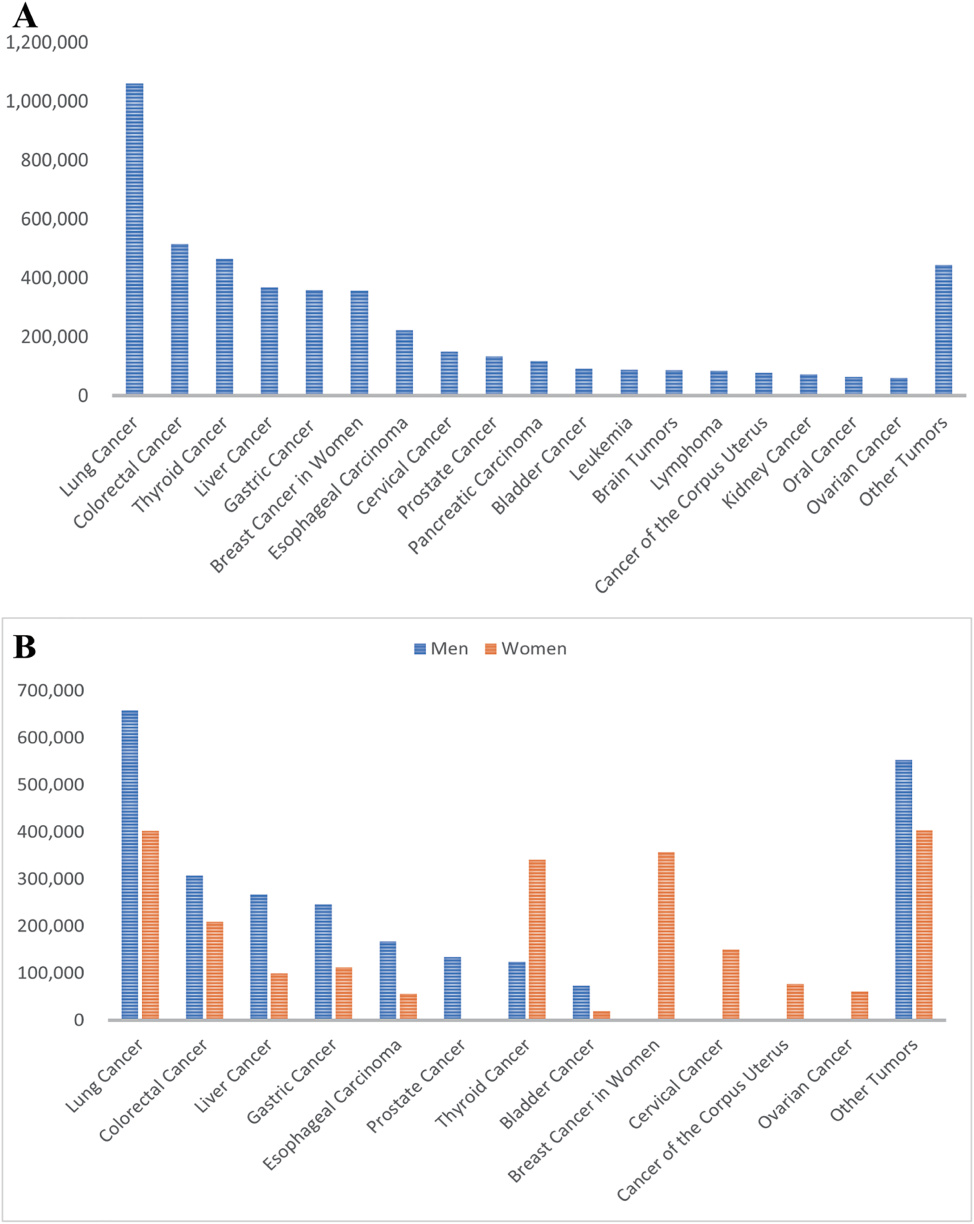
Statistics on the incidence of malignant tumors in China in 2022. (A) Histogram of the number of malignant tumor cases in China in 2022. (B) Comparison of the number of malignant tumor cases in China by sex in 2022.

**Figure 2:**
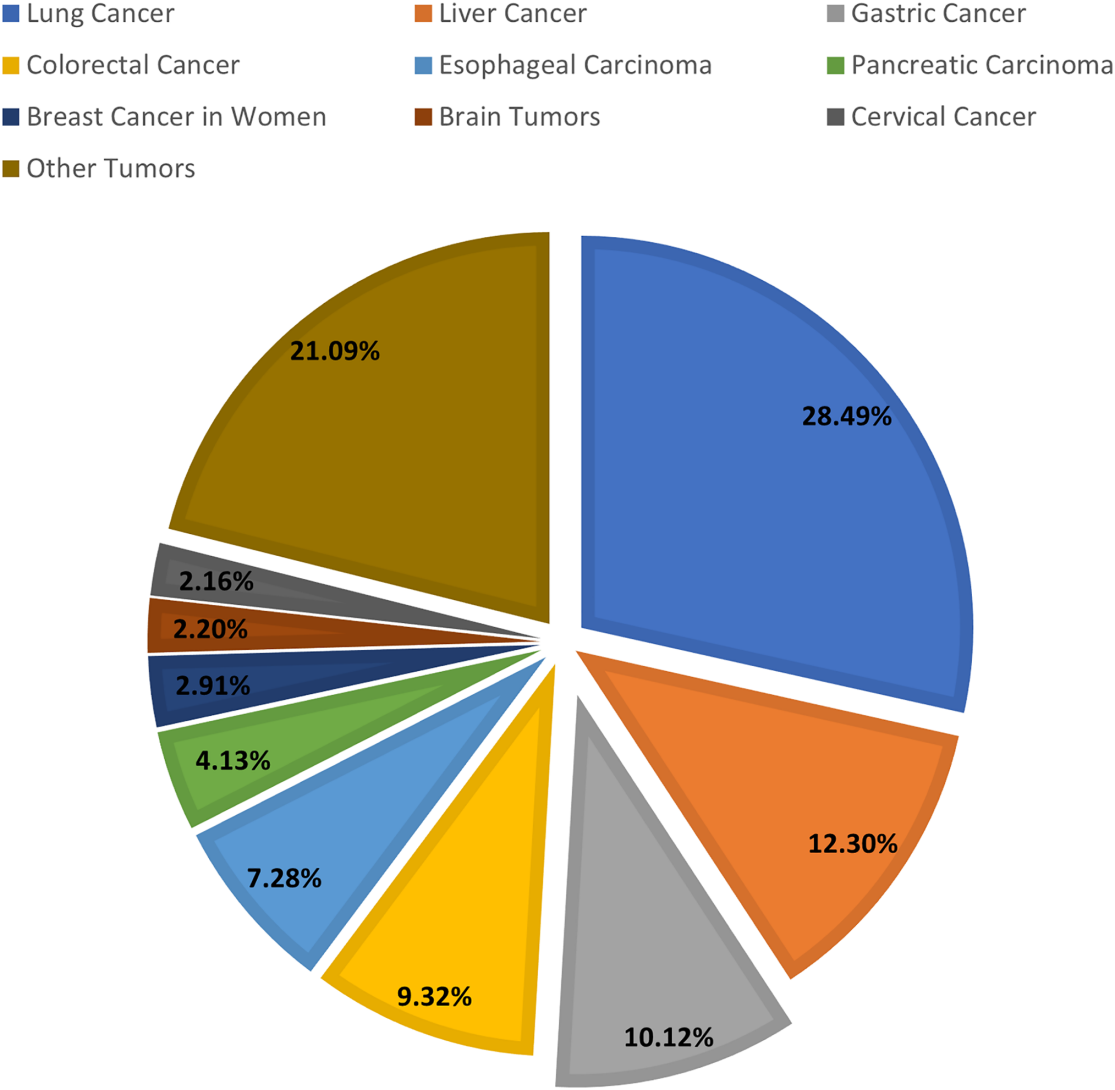
Statistics on the number of malignant tumor deaths in China in 2022. Other tumors include oral cancer, nasopharyngeal cancer, gallbladder cancer, laryngeal cancer, bladder cancer, and other malignant tumors, and the proportion of deaths from each malignant tumor in the total number of deaths from malignant tumors is lower than that of gastric cancer.

Both the incidence and mortality rates of stomach cancer are much higher in China than the global average, making it one of the countries with the highest incidence rates worldwide, which may be related to the low prevalence of dietary structure, environmental factors, and screening. According to the latest analysis of the prevalence of malignant tumors in China, the incidence of gastric cancer ranks fifth among the incidence of malignant tumors in China and third among digestive tract tumors (Fig. 1A) [2]. In line with the worldwide sex disparity in the incidence of gastric cancer, the incidence of gastric cancer among men in China is ranked fourth, and it is 1.52 times that of women (Fig. 1B). This consistency is mainly related to the higher rates of smoking and alcohol consumption in men, and the biological response to H. pylori infection. Gastric cancer accounts for 10.1% of all malignant tumor deaths in China, ranking third (Fig. 2) [2].

**Figure 3:**
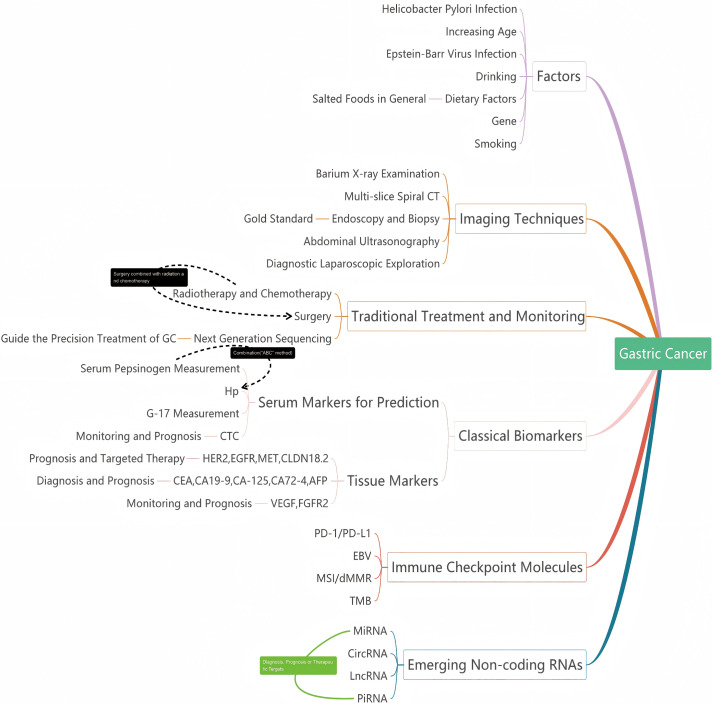
The pertinent variables, diagnostic techniques, and associated biomarkers in the incidence and progression of cancer of the gastric tract. Classical serum and tissue markers in gastric cancer, associated with early diagnosis, gastric cancer classification, immunotherapy, and prognosis, as well as emerging non-coding RNAs with potential clinical applications. CT, computed tomography; GC, gastric cancer; Hp, Helicobacter pylori; G-17, gastrin-17; CTC, circulating tumor cell; HER2, human epidermal growth factor receptor 2; EGFR, epidermal growth factor receptor; MET, mesenchymal-epithelial transition factor; CLDN18.2, Claudin 18.2; CEA, carcinoembryonic antigen; CA19-9, carbohydrate antigen 19-9; CA-125, carbohydrate antigen 125; CA72-4, carbohydrate antigen 72-4; AFP, alpha-fetoprotein; VEGF, vascular endothelial growth factor; FGFR2, fibroblast growth factor receptor 2; PD-1/PD-L1, programmed cell death protein 1/programmed death-ligand 1; EBV, Epstein-Barr virus; MSI/dmmR, microsatellite instability/ mismatch repair deficiency; TMB, tumor mutational burden. MiRNA, MicroRNA; CircRNA, circular RNA; LncRNA, long non-coding RNA; PiRNA, PIWI-interacting RNA.

In this review, the following aspects are discussed: (1) variables that contribute to the development of stomach cancer; (2) precancerous screening, diagnosis, and comprehensive treatment of gastric cancer; (3) classical biomarkers and immune molecular checkpoints in gastric cancer; (4) emerging non-coding RNAs with potential clinical therapeutic prospects (Fig. 3).

## Gastric Cancer

### Factors of gastric cancer

The main factors of gastric cancer include Helicobacter pylori (H. pylori) infection, increasing age, Epstein-Barr virus (EBV) infection, dietary factors, genetic factors, smoking, and others [3–7]. Classified by the anatomical subsite of the stomach, gastric cancer mainly falls into two categories: cardia gastric cancer and non-cardia gastric cancer. The vast majority of non-cardia gastric cancers can be attributed to chronic infection with Helicobacter pylori [8,9]. In addition, smoking, drinking, and eating preserved foods (including salted vegetables, fish, and salted foods in general) increase the risk of non-cardia stomach cancer [10].

The correlation between Helicobacter pylori and cardia carcinogenesis is related to regional factors. Gastric cancer studies in European and American populations and Australian populations generally report that there is no clear association between Helicobacter pylori infection and the occurrence of cardia cancer [11], Gastric cancer studies in populations in East Asian countries often show that both non-cardia gastric cancer and cardia cancer are highly associated with Helicobacter pylori infection in populations in East Asian countries [12]. In Asian populations, the population-attributable fraction of cardia cancer caused by Helicobacter pylori infection was 60.7%, and the population attributable fraction (PAF) of non-cardia cancer was 71.2%. Helicobacter pylori infection caused non-cardia carcinoma in European populations was 73.2% [13]. The prevention and early eradication of Helicobacter pylori is an effective means to reduce the incidence of gastric cancer. By improving dietary hygiene, strengthening screening, and standardizing treatment, combined with the support of public health policies, it is conceivable to drastically lower the burden of H. pylori infection and the problems it causes. Cardia carcinoma in European and American populations is more similar to esophageal adenocarcinoma, which often occurs distal to the esophagus and gradually affects the non-atrophic gastric cardia mucosa, and the predisposing factor is likely to be gastroesophageal reflux disease (GERD) [6,14].

In recent years, while the age-standardized incidence of gastric cancer in China has exhibited a notable downward tendency, its mortality rate remains high. The elevated mortality rate of gastric cancer is primarily associated with its unfavorable prognosis. Early gastric cancer does not have typical clinical symptoms, and most patients are even asymptomatic in the early stage. The most common symptoms of mucosal-associated lymphoid tissue (MALT) gastric marginal lymphoma are usually non-specific gastritis or peptic ulcers under endoscopic examination, and space-occupying lesions are rare, making early diagnosis difficult [15]. At the same time, gastric cancer has strong metastasis and invasion ability, resulting in the diagnosis of gastric cancer patients with intermediate and advanced disease courses accompanied by distant metastasis of varying degrees. At this time, it is difficult to control the development of advanced gastric cancer through surgery, radiotherapy chemotherapy, and other treatment methods, the prognosis is poor and prone to recurrence, and the quality of life of patients is low, resulting in a short survival cycle, and the five-year survival rate is usually 30%–35% [3]. EBV is present in tumor cells in about 10% of gastric cancer patients, and according to statistics, EBV-positive gastric cancer patients have a longer survival time and a higher survival rate [16].

### Histological types of gastric cancer

According to the histological type, Lauren proposed to divide gastric cancer into “gut” subtypes and “diffuse” subtypes, and this classification scheme is widely used [17]. Intestinal gastric cancer is the most common subtype of gastric cancer with glands or tubules resembling intestinal mucosal epithelium, scattered goblet cells, and there are links between cells, mainly occurring in the antrum, and there is a high risk of liver metastasis [18,19]. The occurrence of gastrointestinal cancer is related to the combination of pyloric screw infection, diet, and environment [20]. In the diffuse gastric cancer type, there is no adhesion between the cells and poor cell differentiation, and a single gastric cancer cell or a small subset of cancer cells with a signet ring or non-signet ring form extensively infiltrates the gastric wall [19,21]. Gastritis is linked to diffuse gastric cancer, which occurs in the gastrointestinal mucosa. Studies have shown that the DNA methylation level of gastric mucosa is closely related to H. pylori-associated gastritis, which may be a molecular mechanism for the occurrence of diffuse gastric cancer [22].

**Figure 4:**
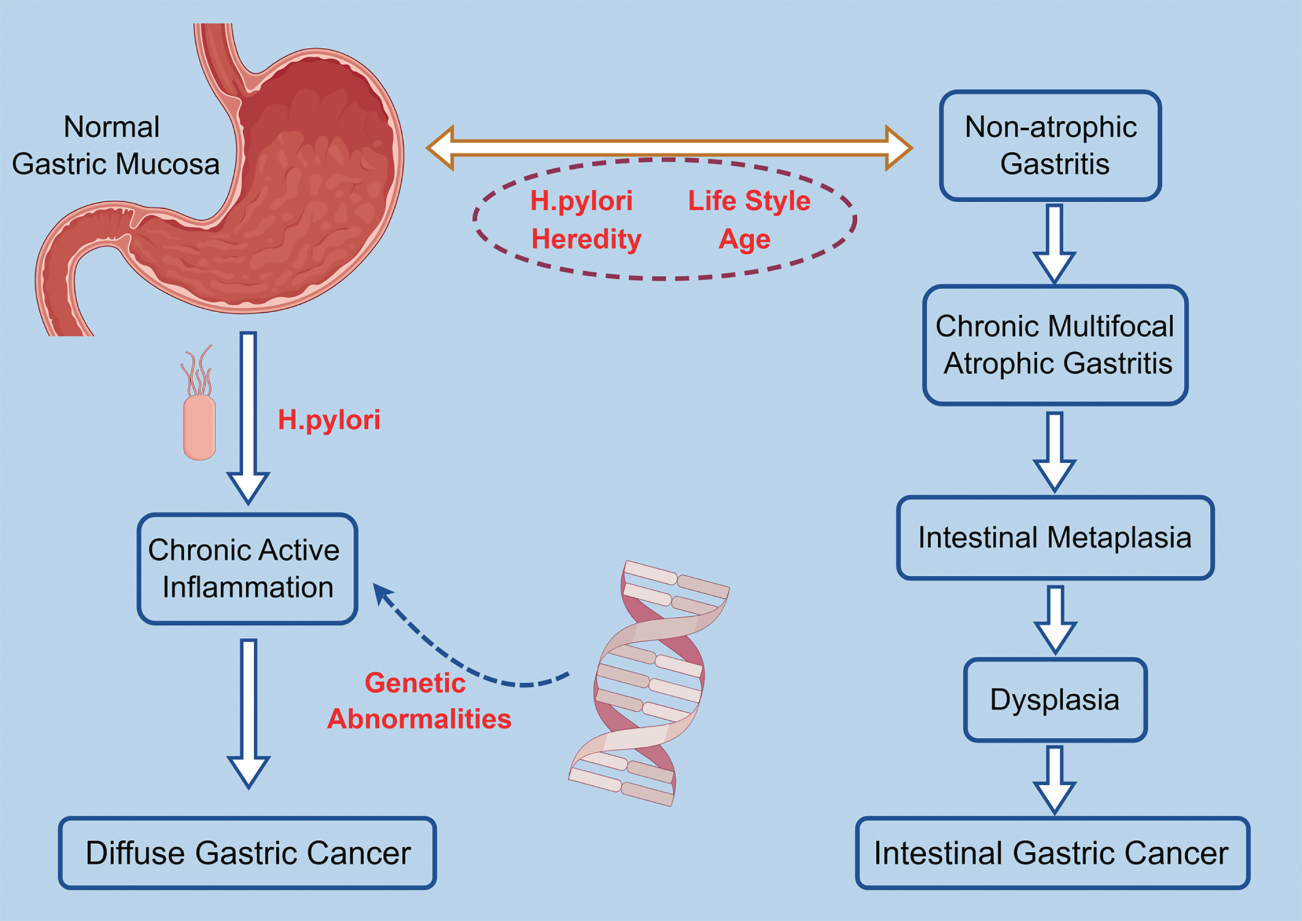
The Correa cascade hypothesizes the gradual transformation of normal gastric mucosal cells into malignant tumors, progressing from normal gastric mucosal infection with *Helicobacter pylori* to non-atrophic gastritis, chronic atrophic gastritis, intestinal metaplasia (precancerous lesions), dysplasia, and finally, enteric gastric cancer. The occurrence of diffuse gastric cancer may be related to *Helicobacter pylori* infection, which is a direct result of chronic active gastritis [23,24]. Fig. 4 was created by figdraw.com.

A form of histological alteration of the gastric mucosa that is predisposed to cancer is referred to as a precancerous lesion of gastric cancer, mainly including gastric mucosal atrophy, intestinal metaplasia (IM), and intraepithelial neoplasia (IN) (Fig. 4).

## Screening and Diagnosis of Gastric Cancer

### The gold standard of diagnosis

At present, the gold standard for diagnosing gastric cancer is endoscopy and biopsy [25], which has a higher detection rate than other tests and can detect ultrastructural lesions and non-ulcerative lesions that may be missed by conventional barium examination [26]. Allowing the doctor to directly and clearly observe the condition of the stomach wall and mucous membranes and perform a pathological biopsy of the suspicious lesion to confirm the diagnosis further [27].

Gastroscopy is an invasive procedure that causes patients to suffer a certain amount of pain during the examination. Moreover, the cost of endoscopy is relatively high, which makes the acceptance of gastroscopy not high among patients. In recent years, painless gastroscopy has developed rapidly, but due to its high cost, it is not suitable as a universal screening method. Thus, a more popular approach in clinical practice is to screen for stomach cancer in high-risk individuals, initially using non-invasive examination and then taking the gastroscopic precision examination to confirm the diagnosis.

### Screening of precancerous lesions

Screening serves as a crucial approach for enhancing the detection rate of early-stage gastric cancer. By doing so, it can effectively enhance the quality of life and extend the survival period of gastric cancer patients. In China, the incidence rate of gastric cancer ranks third among digestive tract tumors, and its mortality rate also occupies third place among all malignant tumors. The majority of gastric cancer patients are diagnosed at an advanced stage, which leads to a low five-year survival rate, poor prognosis, and a high recurrence tendency. Despite this, there is still no corresponding large-scale gastric cancer screening program in China due to high cost, poor public acceptance, and low availability. Through the development of screening guidelines, the promotion of non-invasive screening methods, health education, and the establishment of a multi-level screening network, large-scale gastric cancer screening programs can be effectively implemented, and public acceptance can be improved. This will not only help to detect and treat gastric cancer early but also significantly reduce the incidence and mortality of gastric cancer and improve the health level of the whole population.

Non-invasive or minimally invasive primary screening methods for gastric cancer mainly include Helicobacter pylori detection, serum marker monitoring, and novel biomarker detection, but they cannot be used for diagnosis alone, and their sensitivity and specificity are limited, and they need to be combined with endoscopy or other diagnostic methods to confirm the diagnosis.

#### Clinical manifestations

In the early stage of gastric cancer, it usually presents with non-specific symptoms or no symptoms, and with the progression of the disease, symptoms similar to gastritis and gastric ulcers may appear: indigestion, satiety after eating, loss of appetite, acid reflux, nausea, vomiting, black stool, and other symptoms, which are difficult to pay attention to.

When gastric cancer develops to an advanced stage, in addition to the above non-specific manifestations, there will also be (1) weight loss; (2) persistent stomach pain; (3) progressive dysphagia and reflux can be seen in cardia cancer; (4) jaundice. Severe wasting, anemia, edema, and cachexia may be seen in advanced gastric cancer [28].

Patients with early-stage gastric cancer usually have no obvious signs, and patients with advanced and advanced gastric cancer may have the following signs: (1) deep tenderness in the upper abdomen; (2) abdominal mass; (3) when pyloric obstruction occurs, gastric type and gastric water shaking sound can be seen; (4) ascites signs can be seen when peritoneal metastasis occurs; (5) supraclavicular fossa lymphadenopathy, and others.

Although the diagnosis of gastric cancer cannot be directly based on the above clinical manifestations, and the positive predictive value of clinical manifestations for gastric cancer is less than 10% [29], the combination of relevant clinical manifestations and signs still has important reference value for the diagnosis and differential diagnosis of gastric cancer.

#### Imaging techniques

Gastrectomy is the treatment option for the vast majority of gastric cancer patients, and appropriate and correct surgery and treatment planning are related to accurate Tumor Node Metastasis (TNM) staging. In the process of staging and diagnosing gastric cancer, different imaging detection methods are often used.

Upper GI angiography (Barium X-ray examination) can indirectly observe the shape, size, mucosal texture, wrinkle state, position, and peristalsis of the stomach, and can detect signs such as small diameter of the gastric cavity, narrowing, deformity, stiffness, niches, filling defects, swelling of the gastric wall, and polypoid lesions [30]. Since the positive rate and accuracy of barium studies are lower than those of gastroscopy [6,31], and it is easy to miss the diagnosis when small lesions occur, it is recommended to combine the results of other examinations for combined testing.

The main option for the clinical stage of TNM in gastric carcinoma is multi-slice spiral Computed Tomography (CT), which may assess the condition of the stomach wall and cavity and identify any metastases or invasion of cancer cells in the perigastric organs and lymph nodes. Multi-phase contrast-enhanced scans and multi-plane reconstruction images can more accurately determine the tumor location and the relationship between the tumor and surrounding organs or blood vessels and distinguish the tumor from the regional lymph nodes. The overall accuracy of CT scans after multi-planar reformation (MPR) images was 82% for T staging, and 76%–85% for T staging. The staging ability of preoperative lymph nodes (LN) was poor, with an overall accuracy of 66%. The overall accuracy of preoperative M staging for gastric cancer was 82% with C4 detectors (CT) (scanners with ≥4 detectors) [32].

When CT scan results suggest that gastric cancer may metastasize to distant sites, Positron Emission Tomography-Computed Tomography (PET-CT) is recommended to evaluate the patient’s systemic condition. It is not recommended for diffuse gastric cancer and mucinous adenocarcinoma [33]. For advanced gastric cancer, the detection rate of PET reaches 83%–100%, but only 26%–63% for early-stage gastric cancer; Although the specificity of fluorodeoxyglucose (FDG)-PET increased from 62% to 92% compared with CT, the sensitivity of the detection of regional lymph node metastases decreased from 78% to 53% [34]. Studies have shown that the combination of FDG-PET and CT imaging can significantly improve the accuracy of the preoperative staging of gastric cancer [35]. The combined detection of PET-CT can be used to evaluate the preoperative M stage of gastric cancer with maximum accuracy. Contrast-enhanced magnetic resonance imaging (MRI) is recommended when liver metastases from gastric cancer are suspected [36]. The overall accuracy of MRI in the diagnosis of preoperative T staging was 83%, and the staging accuracy was 77% to 87%.

Abdominal ultrasonography (AUS) is a non-invasive and radiation-free diagnostic method that can examine the location, size, and shape of gastric wall tumors and can also preliminarily understand the metastasis of perigastric organs. However, transabdominal ultrasonography has a low detection rate of gastric cancer and is only used as a supplementary examination.

The gold standard for identifying stomach cancer at this time is a gastroscopy and a gastroscopic biopsy. The T and N stages of stomach cancer can be diagnosed by endoscopic ultrasonography (EUS). Studies have shown that EUS has a sensitivity and specificity of 86% and 90% for primary tumors T1-T2 (superficial) and T3-T4 (advanced), and 83% and 67% for N stage lymph node metastases respectively [37,38], with an accuracy not lower than that of CT.

For patients with locally advanced disease, especially those with high-risk factors for peritoneal metastases who are planned to receive preoperative therapy, diagnostic laparoscopic exploration is advocated. This approach can help detect occult metastases.

## Integrative Treatment and Monitoring

According to the classification and staging of the tumor, combined with the general condition of the patient and the functional status of the organs, the multidisciplinary team (MDT) treatment model is adopted, and the principle of individualization and standardization is followed.

### Radiotherapy and chemotherapy

Patients with advanced metastatic gastric cancer who have no chance of surgery are treated with comprehensive treatment based on drug therapy, and the concept of MDT is implemented in the treatment process. Drug treatment mainly consists of chemotherapeutic agents, molecularly targeted medications, and immune checkpoint inhibitors.

Clinically, chemotherapy drugs remain the cornerstone of treatment for advanced gastric cancer, and European Society for Medical Oncology (ESMO) guidelines recommend chemotherapy in the perioperative period [39]. Studies have shown that in an overall survival analysis, chemotherapy-assisted surgery is associated with a 27% to 29% reduction in mortality compared with surgery alone [40], chemotherapy at 6 to 8 weeks postoperatively may prolong survival to some extent [41], possibly because postoperative chemotherapy reduces the likelihood of locoregional recurrence by reducing nodal, local, and peritoneal recurrence [42]. Meta-analyses indicated that, in comparison to the postoperative chemotherapy group and the surgery-only group, the neoadjuvant chemotherapy group demonstrated a significantly higher 1-year and 3-year survival rate. Moreover, the 5-year survival rate of the neoadjuvant chemotherapy group was markedly superior to that of the surgery-only group [43].

In addition to diffuse gastric cancer, patients with gastric cancer who underwent D2 surgery with adjuvant chemotherapy combined with radiotherapy had significantly longer survival and a 20% lower mortality rate than those in the single surgery group [44]. In the case of gastric cancer patients, when compared with adjuvant chemotherapy, adjuvant chemoradiotherapy can notably enhance the 5-year disease-free survival (DFS) and decrease the local-regional recurrence rate (LRRR) [45]. Regarding gastric cancer patients with lymph node metastases, adjuvant chemoradiotherapy (CTRT) is associated with a longer survival duration as opposed to adjuvant chemotherapy [46]. Adjuvant chemoradiotherapy is not recommended for patients undergoing neoadjuvant chemotherapy, as studies have shown no significant advantage of postoperative chemoradiotherapy over postoperative chemotherapy [47].

### Next-generation sequencing: NGS

Next-generation sequencing (NGS), also known as massively parallel sequencing, is a breakthrough in genomic research, guiding personalized medicine (PM) by being able to detect somatic driver mutations, mechanisms of resistance, quantification of mutation burden, germline mutations and laying the foundation for new approaches to cancer treatment [48,49]. NGS can be used to evaluate the genetic changes of gastric cancer, which can guide the precision treatment of gastric cancer. Genomics coupled with NGS has been used to identify novel therapeutic targets for gastric cancer [50].

### Gene therapy

Gene therapy for gastric cancer mainly uses gene editing, gene silencing, or gene enhancement to regulate abnormal genes associated with gastric cancer, thereby inhibiting tumor growth or enhancing the immune system’s ability to fight cancer.

Clustered regularly interspaced short palindromic repeat (CRISPR) and CRISPR-associated protein9 (Cas9) are breakthrough technologies in genetic research. CRISPR-Cas9 can knock in or knock out several genes at the same time to delete oncogenes or restore the function of tumor suppressor genes. In gastric cancer, CRISPR-Cas9 is used to construct organoids with corresponding genes knocked out and to screen key genes related to the occurrence, metastasis, and chemotherapy resistance of gastric cancer [51].

An oncolytic virus is a genetically modified virus (adenovirus, herpes, etc.) that selectively infects and kills cancer cells while causing an anti-tumor immune response [52,53]. The targeting and immune effects of oncolytic virus therapy make it have unique advantages in the treatment of gastric cancer, but it is still in the experimental stage, and further large-scale clinical trials are needed, combined with gene editing, personalized virus design, and other schemes, to achieve personalized precision medicine for gastric cancer.

CAR (Chimeric Antigen Receptor)-T cell therapy is still in the experimental stage in gastric cancer, and CLDN18.2-CAR-T (CT041) has shown promising treatment potential for gastric cancer [54]. The experimental results suggest that CAR-T therapy may become an important treatment modality for patients with advanced gastric cancer. Possibly due to the limited expression of CLDN18.2 in normal tissues, CT041 has a higher safety profile than HER-2-CAR-T and has good efficacy in patients with diffuse metastatic gastric cancer [55].

With the advancement of gene editing technology, gene therapy is expected to become an important means of personalized treatment for gastric cancer. However, gene therapy still faces problems such as low vector delivery efficiency, off-target effects, immunogenicity, and drug resistance. Currently, the majority of gene therapies for gastric cancer are either in the laboratory phase or the early clinical trial stage and have not been extensively applied in clinical settings. However, some studies have shown its potential to inhibit tumor growth and improve prognosis. In the future, gene therapy may be combined with traditional therapies (e.g., chemotherapy, radiotherapy) to form more effective comprehensive treatment options.

## Blood Tests and Biopsies

### Serum markers

Serological examinations mainly include serum pepsinogen to detect gastric function, gastrin-17 (G-17) to evaluate gastric atrophy, and the detection of multiple tumor markers.

Serum pepsinogen measurement is a serological screening method for gastric cancer. Serum pepsinogen (PG) includes PGI and PGII. As the gastric fundic gland mucosa sheds, the concentration of PGI decreases while the concentration of PGII does not change. Therefore, a low concentration of PGI or low PGR (PGI/PGII ratio) indicates the occurrence of atrophic gastritis (intestinal gastric precancerous lesions). The common cut-off value for defining atrophic gastritis in Japan is “PGI < 70 ng/mL and PGR < 3.0” [31]. Studies have shown that low concentrations of PGI or low PGR are associated with gastric carcinogenesis [56]. Moreover, PGR can serve as a continuous indicator for gastric carcinogenesis. This implies that the lower the PGR level, the greater the risk of gastric cancer [57]. Abnormal levels of PG can serve as a screening tool for the high-risk population of gastric cancer. However, its limitation lies in the fact that it is influenced by gastric mucosal inflammation and atrophy. Therefore, it is merely employed as an auxiliary examination method.

Since serum pepsinogen detection indicates the occurrence of atrophic gastritis, it is more suitable for screening for intestinal gastric cancer.

The integration of Helicobacter pylori serology and serum pepsinogen concentration can contribute to more accurately predicting the incidence and development of gastric cancer (Table 1). The risk of gastric cancer is highest when the PGI is low (or low PGR) and H. pylori antibody is negative, which may be due to a decrease in the number of H. pylori due to severe gastric atrophy [58,59].

**Table 1:** The ABC method assesses the risk of gastric cancer

PG	Hp	Risk of GC
(−)	(−)	+
(−)	(+)	++
(+)	(+)	+++
(+)	(−)	++++

In Table 1, the integration of serum PG and Hp antibody (the “ABC” approach) is employed to evaluate the risk of gastric cancer and screen high-risk populations for gastric cancer: “PGI ≤ 70 μg/L and PGR ≤ 3.0” is defined as PG positive (PG+), and “serum Hp antibody titer ≥30 U/mL” is defined as Hp positive (HP+) [23].

Gastrin-17 (G-17) is a peptide hormone produced by G cells in the antrum. It regulates the secretion of gastric acid and pepsin. The concentration of G-17 is related to the number of G cells in the antrum and gastric acid, and there is a feedback pathway that controls gastric acid and pepsin in the gastrin link, and the concentration of G-17 decreases when atrophic gastritis destroys the inhibition of the feedback pathway or the gradual loss of G cells with the aggravation of atrophy and the high gastric acid in the patient [60]. Studies have shown that when the gastric body atrophies and the antrum is relatively intact, the gastric acid concentration decreases, and the G-17 concentration increases. When atrophy occurs only in the antrum, serum G-17 concentrations are significantly lower than in patients without atrophic gastritis [61]; when atrophy takes place in both the antrum and body, G-17 levels can be either normal or low [58]; G-17 levels are markedly elevated in patients with stomach cancer [26]. The determination of G-17 concentration can be used as an indicator of the morphological status of the gastric antral mucosa, but cannot be used as a single serum marker for gastric cancer alone.

Since changes in G-17 concentrations are not only associated with atrophic gastritis but also affected by gastric acidity and H. pylori infection [62], the consideration of G-17 as a biomarker for precancerous lesions in gastric cancer should be accompanied by the combination of PGI (or PGR) and H. pylori infection.

Studies have shown that low G-17, low PGI, and low PGR can be used as detection indicators for atrophic gastritis, and low PGI, low PGR, and high G-17 can be used as screening indicators for atrophic gastritis [63]. The risk of gastric atrophy and gastric cancer was assessed after the levels of PGI (PGR) and G-17 were detected by serological tests, and gastroscopy was recommended for high-risk patients to improve the detection rate of early gastric cancer.

### Classical tumor biomarkers

Currently, the detection of tumor biomarkers assumes a crucial significance in the clinical diagnosis, classification, and molecular characterization of gastric cancer. Given the relatively low sensitivity and specificity of single markers in gastric cancer diagnosis, a combination of multiple tumor markers is frequently employed to enhance the positive detection rate. The combined detection of tumor markers can dynamically observe the occurrence and development of tumors, the evaluation of clinical efficacy and prognosis, and the possibility of tumor progression or recurrence in time, thereby improving the detection rate and differential diagnosis accuracy of gastric cancer, and can play a certain role in clinical diagnosis and prognosis. Classification based on histological subtype and molecular characteristics can help improve early diagnosis rates and have an impact on treatment.

Prior research has indicated that cancer embryo antigen (CEA) may serve as an independent risk factor associated with a poor prognosis in early gastric cancer. Moreover, patients presenting with carbohydrate antigen 19-9 (CA19-9) positivity frequently exhibit lymph node metastasis. However, the sensitivity and specificity of these indicators in gastric cancer are low, for example, the positive rate of CEA in gastric cancer is only 4.3%, the positive rates of CA19-9, glycohydrate chain antigen 72-4 (CA72-4) and carbohydrate chain antigen (CA-125) are 1.5%, 4.8% and 1.9%, respectively, and the positive rate of the combined detection of the above indicators is only 10.4% [64]. CA-125 has certain diagnostic and prognostic values for peritoneal metastasis and AFP for special pathological types of gastric cancer. The specificity and sensitivity of these protein molecules for gastric cancer diagnosis still need to be further analyzed. Studies have shown that the biomarkers that can be used for early diagnosis or prognosis of gastric cancer include “human epidermal growth factor receptor 2” (HER2) [65], “Hepatocyte growth factor receptor” (HGFR) [66], “Fibroblast growth factor receptor” (FGFR2) [67], “Epidermal growth factor receptor” (EGFR) [68], “Claudin18.2” (CLDN18.2) [69], and others.

#### Human epidermal growth factor receptor 2 (HER2)

HER2 is encoded by the oncogene ERBB2, and studies have shown that the activation of HER2 mutations is an important pro-cancer factor. The main mechanism of activation is that the amplification of the HER2 gene leads to complete overexpression of the Her2 protein on the cell membrane [70]. Approximately 10%–20% of all gastric cancers exhibit HER2 positivity. Currently, there is a growing trend in the development and application of anti-HER2 drugs. These medications aim to enhance the survival rate and prognosis of patients with HER2-positive advanced gastric cancer [71]. For detecting HER2 overexpression in patients with advanced gastric adenocarcinoma, immunohistochemistry (IHC) and fluorescence *in situ* hybridization (FISH) are suggested [72].

Based on the observations of HER2 and its significant intratumoral heterogeneity, multiple biopsy fragments from the site of metastasis of the primary tumor or resection of the primary tumor are required. In the case of biopsy specimens, the current protocol recommends the need for a minimum of 5 biopsy specimens, preferably 6 to 8 biopsy specimens to interpret heterogeneity within the tumor and provide the necessary tumor material for diagnosis and biomarker testing [73].

#### Vascular endothelial growth factor (VEGF)

In the course of gastric cancer, VEGF plays a significant role in encouraging the growth of endothelial cells, as well as inducing carcinogenesis and tumor angiogenesis [74]. The study found that the positive rate of VEGF in gastric cancer tissues was about 52.1%, which was significantly higher than that of adjacent tissues by 16.8% [75], which was related to the growth, invasion, metastasis, and spread of gastric cancer, clinical stage and poor prognosis [76]. VEGF can be a predictor of tumor recurrence and can be used as a good biomarker for disease progression or remission during gastric cancer surveillance, but it cannot be used as an independent clinical diagnostic factor, and can only play an auxiliary diagnostic role [77].

#### Circulating tumor cell (CTC)

After extensive surgery, circulating tumor cells, which are tumor cells breaking away from solid tumor tissue and entering the peripheral blood circulation, are considered to be early signs of tumor recurrence. The presence of CTC is associated with poor chemotherapy response in patients with metastatic gastric cancer, and the survival of CTC-positive gastric cancer patients is significantly shorter than that of CTC-negative patients [78]. The existence of CTC serves as an independent unfavorable factor for postoperative recurrence in gastric cancer patients. It is correlated with advanced tumor stages, peritoneal metastasis, and a shorter survival period [79,80]. It has been shown that the assessment of CTC in peripheral blood may be effective in predicting tumor progression, prognosis, and chemotherapy response in patients with gastric cancer [81].

#### Epidermal growth factor receptor (EGFR)

Studies have shown that overexpression of EGFR often occurs in gastric cancer and promotes chemotherapy resistance in cancer cells, which is associated with poor prognosis [82]. EGFR-mediated MAPK and PI3K pathways are still activated after chemotherapy because the α α-subunit of glycoprotein hormones (CGA) of N-glycosylated glycoprotein hormones binds to EGFR and activates EGFR signaling, promoting the proliferation and development of cancer cells [83].

#### Fibroblast growth factor receptor (FGFR2)

FGFR2 possesses tyrosine kinase activity and is crucial for cell proliferation and the induction of angiogenesis. It achieves this by activating the MAPK, PI3K, STAT, and phospholipase Cy signaling pathways. Studies have shown that FGFR2 amplification occurs in gastric cancer with a probability of 3%–16%, and FGFR2 amplification is associated with advanced diffuse gastric cancer and a poorer prognosis [84].

#### Hepatocyte growth factor receptor (HGFR)

The mesenchymal-epithelial cell transition factor receptor (MET) is a receptor for hepatocyte growth factor (HGF) that can be amplified in gastric cancer [85]. Overexpression of MET is associated with a more aggressive phenotype, i.e., advanced local tumor growth, lymph node spread, distant metastases, advanced tumor stage, recurrence, and low survival [86]. The overexpression rate of MET in gastric cancer is 58%, but it is not recommended as a separate prognostic marker for gastric cancer [87]. Until now, there has been no standardized MET scoring system for gastric cancer. In addition, like HER2, MET shows intratumoral heterogeneity: expanded and unexpanded tumor cell clones occur in the same tumor and are distinguishable at the cellular level [88–90], further weakening its use as a predictive biomarker.

#### Claudin18.2 (CLDN18.2)

It was found that CLDN18.2 was abnormally expressed in the occurrence and development of gastric cancer, and was involved in the proliferation, differentiation, and invasion of tumors. The expression of CLDN18.2 was significantly elevated in diffuse histological subtypes of gastric cancer and high-grade tumors [69]. CLDN18.2 is a highly selective labeled protein that is expressed only in differentiated gastric mucosal epithelial cells. Due to its unique expression pattern, CLDN18.2 has become a unique molecule for targeted therapy for gastric cancer [91]. Research has indicated that Cldn18.2 is related to tumor immune infiltration, the PD-1 pathway, the cell cycle, and the Wnt signaling pathway [92].

## Immune Checkpoint Molecules

Given that advanced gastric cancer is characterized by a dismal prognosis and a low survival rate, the development of more effective treatment strategies to prolong patients’ lives has become an urgent necessity. Immunotherapy has currently emerged as an innovative approach to treating gastric cancer. This is because it has the potential to reverse immunosuppression and enhance the body’s anti-tumor immune response. Significantly, the immune system plays a pivotal role in regulating the initiation, progression, and metastasis of tumors [93]. Immune cells in the normal human body are able to recognize and eliminate mutant cells to monitor and limit the development of cancer, but cancer cells often multiply rapidly by evading immune surveillance, which is the definition of tumor escape. Tumor-specific antigen (TSA) is an antigen that is expressed only on the surface of tumor cells, and if the immune system ignores the “TSA” on the surface of tumor cells, cancer cells will multiply and spread rapidly. Therefore, we need immunomodulatory antibodies targeting immune cell surface antigens to block immunosuppressive signals and enhance immune cell activity, so as to achieve the purpose of supplementing or activating T cells and reversing immunosuppression [94].

The implementation of immune checkpoint inhibitors (ICIs) has brought about substantial alterations in the treatment of diverse cancers. However, the long-term effects include potential immune-related toxicity, drug resistance issues, and impact on quality of life, requiring individualized management and multidisciplinary collaboration. In the future, with the deepening of research and the optimization of treatment strategies, ICIs are expected to play a greater role in the treatment of gastric cancer.

### Programmed death-1(PD-1)

Cytotoxic T lymphocytes (CTLs) and PD-1-mediated immune checkpoint regulation play a crucial role in immunosuppressive mechanisms. In the normal host environment, immune checkpoint molecules modulate T cell responses to antigens by upregulating the costimulatory pathway or downregulating the co-inhibitory pathway of immune signaling [95]. CTLs secrete interferon-γ (IFN-γ) in response to cancer cells, and IFN-γ binds to IFN-γ receptors on tumor cells, thereby stimulating the JAK-STAT signaling pathway and overexpressing progressive death ligand-1 (PD-L1) [96]. PD-L1 is overexpressed in 40% of advanced gastric adenocarcinoma, but it is undetectable in normal gastric tissue [97]. PD-1 is a small immune checkpoint molecule that is ubiquitously expressed on T cells and limits T cell activity in peripheral tissues during inflammatory responses, thereby preventing an increase in immune response and autoimmune development [98]. PD-1 is a small immune checkpoint molecule that is ubiquitously expressed on T cells and limits T cell activity in peripheral tissues during inflammatory responses, thereby preventing an increase in immune response and autoimmune development [99]. Due to the poor immunogenicity and complex tumor microenvironment of gastric cancer, only a small proportion of gastric cancer patients have achieved good therapeutic effects with PD-1 inhibitors.

### Epstein-Barr virus (EBV)

Epstein-Barr coding region (EBER) *in situ* hybridization is the gold standard for diagnosing EBV-associated gastric cancer (EBVaGC) [100]. Epstein-Barr virus (EBV) infection can induce an immunocompetent tumor microenvironment, but its relationship with immunotherapy response remains controversial [101,102]. Numerous pieces of evidence suggest the likelihood that Epstein-Barr virus (EBV) infection might take place during the early stage of the pathogenesis in a larger proportion of gastric cancer cases than is commonly assumed. Moreover, the EBV genome has the potential to be randomly incorporated into susceptible regions within the cellular genome. This integration can lead to instability of the host genome or dysregulated gene expression [103].

EBVaGC is a specific subtype of GC that has been found to have lower metastasis rates, better disease-free survival, and a better prognosis than other subtypes of gastric cancer [104], and EBV infection is considered a potential biomarker of GC response to immunotherapy. EBV(+)GC has been shown to be positively correlated with PD-L1 expression overall [105], and patients with EBV(+)GC are more effective than patients with EBV(−)GC who receive PD-1 inhibitors [106]. It has been discovered that EBV infection can enhance the expression of tumor-infiltrating lymphocytes as well as immune checkpoint molecules [107], and the significant accumulation of T and B lymphocytes in EBV(+)GC compared with EBV(−)GC may be the reason why EBVaGC patients are more sensitive to chemotherapy and immunotherapy, and EBVaGC patients are the beneficiaries of immune checkpoint inhibitor therapy.

### Microsatellite instability/deficient mismatch repair (MSI/dMMR)

The reactivity of antibodies that inhibit PD-1 has a direct association with the occurrence of microsatellite instability (MSI). MSI is a crucial genetic subtype of gastric cancer, which is induced by mismatch repair deficiency (dMMR) [108]. Studies have found that MSI-H/dMMR tumor cells can induce endogenous immune anti-tumor responses [109]. The MSI status can be gauged through immunohistochemistry. This involves determining the nuclear expression of proteins associated with DNA mismatch repair (such as MLH1, MSH2, MSH6, and PMS2) to evaluate MMR deficiency; it can also be assessed by PCR, which measures the gene expression levels of microsatellite markers [34]. Patients with MSI gastric cancer are less sensitive to chemotherapy, and immunotherapy for patients with advanced MSI gastric cancer may produce better treatment outcomes if it can be started early. Patients with MSI-H gastric cancer have a higher response rate to ICIs and may achieve longer survival.

### Tumor mutation burden (TMB)

Tumor mutational burden (TMB), defined as the number of somatic mutations per million genome sequences, varies across malignancies [110]. Highly mutated tumors tend to indicate the generation of neoantigens, making them targets for activating immune cells. Microsatellite instability (MSI-H) is often a subset of high TMB, and in gastric cancer, both tend to coexist [111]. Studies indicate that among gastric cancer patients, those with a higher tumor mutational burden (TMB) tend to have a higher survival rate. Specifically, patients with high TMB (TMB-H) enjoy a more substantial survival advantage in overall survival (OS) compared to those with low TMB (TMB-L) [112]. Additionally, gastric cancer patients with greater macrophage invasion might face a poorer prognosis [113].

TMB is a novel biomarker of PD-1 (PD-L1) therapeutic response and may be a predictive biomarker for immunotherapy for gastric cancer [114].

## Non-Coding RNAs with Potential Clinical Application in Gastric Cancer

In recent years, the field of cancer research has placed a great deal of emphasis on non-coding RNAs (ncRNAs). These ncRNAs constitute the majority of the human genome and are transcribed into RNA that doesn’t code for proteins. Studies have shown that they are involved in cell development, proliferation, differentiation, and apoptosis during tumorigenesis, progression, invasion, and metastasis of a variety of cancers, including gastric cancer, and the molecular interacting group composed of ncRNAs and protein is thought to coordinate cancer metastasis [115,116].

According to length, ncRNAs are bounded by 200 nucleotides, with ncRNAs up to 200 nucleotides (nt) defined as small ncRNAs and ncRNAs with more than 200 nucleotides defined as long non-coding RNAs (lncRNAs); according to the functional classification, ncRNAs are generally divided into housekeeping ncRNAs and regulatory ncRNAs. In addition, tRNA-derived fragments (tRFs) have recently been identified as potential biomarkers, but their high-quality clinical studies related to gastric cancer are few and geographically limited [117]. In this review, we focus on the four major ncRNAs in Regulatory ncRNAs, including microRNAs (miRNA), long ncRNAs (lncRNA), circular RNAs (circRNA), and PIWI-interacting RNAs (piRNA) (Table 2) [118,119].

**Table 2:** Functions of regulatory non-coding RNAs in cancers

Symbol	Type of RNAs	Length (nt)	Function
miRNA	microRNAs	22	MiRNAs regulate cancer by degrading mRNA or inhibiting translation
lncRNA	long non-coding RNAs	>200 (Linear)	LncRNAs act as guide molecules to recruit chromatin remodeling factors, as decoys to hinder target gene promoter transcription factors, as sponges for related miRNAs to prevent degradation of target genes, or as scaffolds to promote the interaction of related proteins.
circRNA	circular RNAs	>200 (Circular)	CircRNAs function as miRNA sponges to stop target gene degradation or as scaffolds to help related proteins interact.
piRNA	PIWI-interacting RNAs	24–30	PiRNA plays a crucial role in inhibiting cleavage of transposable factors, deadenylation, and decay.

### MicroRNAs in gastric cancer

Research indicates that miRNAs influence cancer development and advancement by functioning as either tumor suppressors or cancer enhancers. MiRNA is a small non-coding RNA, approximately 22 nucleotides (nts) in length, that regulates the expression of target genes after transcription by binding to the 3’UTR region of the target gene, thereby exerting a variety of biological functions [120].

It has been found that miR-124 can inhibit the proliferation of gastric cancer cells by directly targeting the EZH2 gene, and miR-124 has an additive effect with conventional chemotherapy 5-fluorouracil (5-FU), which has the potential to become a new treatment method for gastric cancer [121]. In addition, miR-124 can target COL4A1 and inhibit the epithelial-mesenchymal transition (EMT) caused by TGF-β1 in GC, thereby inhibiting the development of gastric cancer [122]. MiR-124-3p in the miR-124 family can inhibit the expression of integrin β3 (ITGB3), thereby inhibiting the migration and invasion of GC [123]. The expression level of miR-519a-3p in serum exosomes of gastric cancer patients with liver metastasis was significantly higher than that in patients without liver metastasis, and exo-miR-519a-3p promoted liver metastasis by inducing intrahepatic macrophage M2 polarization to form angiogenesis-rich premetastatic niches, which means that high expression of exosomal miR-519a-3p in gastric cancer patients indicates a poor prognosis [124]. miR-635 is under-expressed in gastric cancer, and its abnormal expression is significantly related to clinicopathological parameters such as increased TNM stage and lymph node metastasis, miR-635 inhibits the proliferation, migration, and invasion of gastric cancer cells by regulating kinesin family member C1 (KIFC1), which plays a role in inhibiting tumors [125]. Research findings indicated that miR-22 had a substantial correlation with the TNM stage and lymph node metastasis of gastric cancer. It might serve as a valuable prognostic indicator for this type of cancer. Moreover, the expression of miR-22 was found to be decreased in gastric cancer. Directly targeting metadherin (MTDH) effectively curbed the invasion and metastasis of gastric cancer cells [126,127]. While enhancing the anti-tumor effects of diallyl disulfide, miR-22 and miR-200b collaborate to rein in the progression of gastric cancer through the Wnt-1 signaling pathway. This indicates that miR-22 and miR-200b might serve as valuable therapeutic targets for gastric cancer [128]. Research has shown that miR-200b and miR-200c could potentially serve as prognostic biomarkers for gauging survival and recurrence in gastric cancer patients. These two proteins are expressed at lower levels in gastric cancer tissues and are associated with the TNM stage and lymph node metastasis in gastric cancer [129–131]. Moreover, miR-200c impacts the onset and development of gastric cancer by modulating EMT [132,133]. MiR-195 is under-expressed in GC, which is associated with high TNM stage, poor cell differentiation, and lymph node metastasis, and miR-195 plays a tumor suppressive role in gastric cancer by regulating G protein-coupled receptor, family C, group 5, member A (GPRC5A) [134]. MiR-665 can target AKT serine/threonine kinase 3 (AKT3) to inhibit GC cell proliferation and promote apoptosis of GC cells [135–137]. The expression of miR-876-5p was reduced in gastric cancer tissues, and its overexpression hindered gastric cancer cell proliferation while encouraging apoptosis. The knockdown of miR-876-5p promotes the proliferation of gastric cancer cells, inhibits apoptosis, and reduces the sensitivity of gastric cancer cells to cisplatin [138]. MiR-19b-3p and miR-215-5p are overexpressed in serum exosomes in gastric cancer patients [139,140], and studies suggest that they can be used as new biomarkers for gastric cancer diagnosis. via the miR-BART5/PIAS3/pSTAT3/PD-L1 axis, miR-BART5-5p directly zeroes in on PIAS3, leading to the upregulation of PD-L1. This exerts an anti-apoptotic impact, spurring the proliferation, invasion, migration, and immune escape of tumor cells. As a result, it facilitates the onset and progression of gastric cancer, especially among the PD-L1(+) cohort of patients with Epstein-Barr virus (EBV)-associated gastric cancer [141]. MiR-185 levels are notably reduced in gastric cancer cases, correlating with both TNM staging and the presence of lymph node metastasis. This suggests that miR-185 could serve as a promising independent biomarker for forecasting survival and recurrence in patients with gastric cancer [142,143].

More and more studies have shown that many miRNAs are specifically upregulated or downregulated in gastric cancer. MiRNAs play a role in promoting or suppressing cancer by inhibiting the expression of target genes. Some of these target genes are directly or indirectly involved in some typical tumor signaling pathways. Even though research on miRNA in gastric cancer is still in its early days, the role of miRNA in the development and onset of gastric cancer is clear. MiRNA analysis techniques are considered to be a useful means for the early detection and prognostic prediction of gastric cancer. The investigation of miRNA in human gastric cancer is crucial for advancing diagnostic and treatment strategies [139].

We summarize some studies on the function of miRNAs in gastric cancer in Table 3.

**Table 3:** Functions of non-coding RNAs in GC

Type	Examples	Functions	References
miRNA	miR-124	Inhibit the proliferation of gastric cancer cells	[121–123]
	miR-519a-3p	Induction of premetastatic niche formation promotes liver metastasis	[124]
	miR-635	Inhibit the proliferation, migration, and invasion of gastric cancer cells	[125]
	miR-22	Inhibition of gastric cancer growth, associated with clinical stage and lymph node metastasis	[126–128]
	miR-200b	Inhibition of gastric cancer growth and prognostic assessment	[128–131]
	miR-200c	Inhibition of EMT and prognostic assessment	[129,132,133]
	miR-195	Inhibit the proliferation, migration, and invasion of gastric cancer cells	[134]
	miR-665	Inhibit the proliferation, migration, and invasion of gastric cancer cells	[135–137]
	miR-876-5p	Inhibit the proliferation, migration, and invasion of gastric cancer cells	[138]
	miR-19b-3p	Gastric cancer diagnosis	[139,140]
	miR-215-5p	Gastric cancer diagnosis	[140]
	miR-BART5-5p	Anti-apoptosis, promoting tumor cell proliferation, invasion, migration and immune escape	[141]
	miR-185	Prognostic assessment	[142–143]
circRNA	hsa_circ_006100	Induce malignant transformation of cells	[134]
	circ_15430	Reverse the pro-HP effect on autophagy and inhibit the development of gastric cancer	[147]
	hsa_circ_0015286	Gastric cancer diagnosis and prognostic assessment	[148]
	hsa_circ_100290	Promote gastric cancer proliferation, invasion, and EMT	[145]
	circ-NRIP1	Promote the proliferation, invasion, and metastasis of gastric cancer	[149,150]
	circ-PTPN22	Gastric cancer diagnosis and prognostic assessment	[151]
	hsa_circ_0000745	Associated with differentiation and stage of lymph node metastases	[152]
	hsa_circ_0001789	Related to invasion, differentiation, lymph node metastasis, TNM staging and distant metastasis of gastric cancer	[153]
	hsa_circ_0003159	associated with distant metastasis of gastric cancer and advanced TNM	[154]
	hsa_circ_0006646	Promote EMT	[136]
lncRNA	lncRNA-MIR200CHG	Inhibit EMT	[132]
	lncRNA-LINC00565	Promote the proliferation of gastric cancer and inhibit apoptosis	[135]
	lncRNA-CCAT5	Activate the STAT3 signaling pathway to promote gastric cancer invasion and metastasis	[156]
	lncRNA-TP53TG1	Inhibit the proliferation, metastasis, and cell cycle process of gastric cancer cells; promote apoptosis	[157]
	lncRNA-NEAT1	Promote lymph node metastasis	[158]
	lncRNA-B3GALT5-AS1	Gastric cancer diagnosis and postoperative testing	[159]
	lncRNA-HCP5	Early diagnosis of gastric cancer and the dynamic monitoring of tumors	[160]
	lncRNA-Hoxa11-AS	Promote the proliferation, invasion, and metastasis of gastric cancer	[123]
	lncRNA-SND1-IT1	Promote EMT	[122]
piRNA	piR-823	Inhibit the proliferation, migration, and invasion of gastric cancer cells; prognostic assessment	[162,163]
	piR-651	Promote the proliferation, invasion, and metastasis of gastric cancer; prognosis assessment	[163,164]
	piR-019308	Gastric cancer diagnosis	[165]
	piR-004918	Gastric cancer diagnosis and prognostic assessment	[165]
	piR-018569	Gastric cancer diagnosis and prognostic assessment	[165]
	PIWIL1	Promote the proliferation, invasion, and metastasis of gastric cancer	[166]
	piR-1245	Gastric cancer diagnosis and prognostic assessment	[167]

### Circular RNAs in gastric cancer

Circular RNAs (circRNAs) originate from exons and are predominantly located in the cytoplasm and are defined as a new class of non-coding RNAs with covalently enclosed structures, most of which function as microRNA sponges or regulatory proteins [144], and some circular RNAs containing internal ribosomal entry sites can be translated into polypeptides [145]. CircRNA expression is dynamically controlled in many malignancies and controls the course of cancer via several ways [146].

The expression of hsa_circ_006100 increases during the development of gastric cancer, suggesting a poor prognosis in gastric cancer patients, and hsa_circ_006100 plays a role in promoting cancer by inducing the malignant transformation of gastric cells [134]. Circ_15430 is predominantly located in the cytoplasm of GC cells. It is downregulated in both GC cell lines and tissues and shows a negative correlation with tumor size. Circ_15430 down-regulated expression in HP+ gastritis tissues reversed the effect of pro-HP on autophagy. Down-regulation of circ_15430 can promote the proliferation and migration of GC cells, and inhibit apoptosis and autophagy, and circ_15430 plays an inhibitory role in gastric cancer [147]. Research findings indicated that patients with low levels of hsa_circ_0015286 expression—which is regarded as a diagnostic and prognostic indicator for gastric cancer—had a longer overall lifespan compared to those with high expression. Hsa_circ_0015286 was notably overexpressed in gastric cancer tissues, plasma, and cancer cells. Moreover, it was strongly associated with tumor size, TNM stage, and lymph node metastasis [148]. In GC, the TNM stage, invasion depth, and lymph node metastases were all closely associated with the high expression of Hsa_circ_100290. *In vitro* experiments showed that silencing hsa_circ_100290 led to G0/G1 phase arrest and significantly decreased the viability, colony formation ability, migration capacity, and invasion potential of gastric cancer cells. Hsa_circ_100290 acts as a cancer promoter in gastric cancer to promote cell proliferation, invasion, and EMT [145]. Circ-NRIP1 acts as a sponge of miR-149-5p in gastric cancer to affect the expression level of AKT1 and ultimately as a tumor promoter in gastric cancer [149]. In addition, circ-NRIP1 regulates MYH9 through miR-186-5p to accelerate glycolysis and gastric cancer progression [150]. Circ-PTPN22 is elevated in gastric cancer tissues and shows a positive correlation with metastasis. Studies have shown that the downregulation of circ-PTPN22 can inhibit cell proliferation, migration, and invasion through the epithelial-mesenchymal transition pathway [151]. In the plasma and tissues of gastric cancer patients, Hsa_circ_0000745 showed reduced expression. The level of its expression in the plasma was tied to the stage of tumor-lymph node metastasis. However, in gastric cancer tissues, its expression level was related to the degree of differentiation [152]. Hsa_circ_0001789 was downregulated in gastric cancer tissues and plasma, and was associated with the invasion, differentiation, lymph node metastasis, tumor-node metastasis (TNM) staging, and distant metastasis of gastric cancer [153]. The research discovered that the expression of hsa_circ_0003159 was markedly down-regulated in gastric cancer tissues. Moreover, the expression level of hsa_circ_0003159 was significantly inversely correlated with gender, distant metastasis, and the stage of lymph node metastasis. It may be a potential tumor marker for patients with gastric cancer [154]. In gastric cancer samples,hsa_circ_0006646 exhibited significant expression, which correlated positively with lymph node metastasis, TNM staging, and unfavorable outcomes. Hsa_circ_0006646 targeting the miR-665-HMGB1 axis activated the Wnt/β-catenin pathway to promote the malignant behavior and EMT of GC cells [136].

We summarize recent studies on the function of circRNAs in gastric cancer in Table 3.

### Long non-coding RNAs in gastric cancer

The study found that most long non-coding RNAs (lncRNAs) were microsatellite stable (MSS)/EMT subtype-specific in gastric cancer. LncRNAs play a crucial role in various biological functions, such as cell migration, invasion, proliferation, and apoptosis [155]. LncRNA-MIR200CHG (U47924.27) is a major regulator of EMT in gastric cancer of the MSS/EMT subtype, inducing a mesenchymal phenotype of gastric cancer cells due to MIR200CHG silencing due to hypermethylation of its promoter. Low levels of lncRNA-MIR200CHG expression are likewise associated with EMT characteristics and disease advancement in gastric cancer patients, suggesting a bleak prognosis. MSS/EMT gastric cancer, the most aggressive subtype, is tied to a poor prognosis [132]. LncRNA-LINC00565 is highly expressed in gastric cancer tissues, promotes gastric cancer proliferation, inhibits apoptosis, and is negatively correlated with prognosis [135]. LncRNA-CCAT5 is a direct transcriptional target of the WNT signaling cascade, and lncRNA-CCAT5 is significantly upregulated in gastric cancer and plays a role in promoting the growth and metastasis of gastric cancer in a STAT3-mediated manner, suggesting a poor prognosis [156]. LncRNA-TP53TG1 expression was downregulated in gastric cancer and significantly linked to poor patient survival. LncRNA-TP53TG1 hampers the growth and spread of gastric cancer cells while also interfering with their cell cycle. It encourages apoptosis and serves as a tumor-suppressing factor in both the onset and progression of gastric cancer [157]. LncRNA-NEAT1 reduces regulation of nuclear pre-mRNA domain containing 1B (RPRD1B) ubiquitination by blocking the interaction of RPRD1B with tripartite motif-containing 25 (TRIM25), promoting lymph node metastasis during the development of gastric cancer [158]. The level of lncRNA-B3GALT5-AS1 in the serum of gastric cancer patients is significantly elevated, and its overexpression is significantly associated with lymph node metastasis and TNM staging of gastric cancer [159]. Studies have shown that high expression of serum human histocompatibility complex P5 (HCP5) in gastric cancer is associated with differentiation, lymph node metastasis, and neural invasion. Serum HCP5 is considered to be a new indicator for the early diagnosis of gastric cancer and dynamic monitoring of tumors [160]. LncRNA-Hoxa11-AS overexpressed and down-regulated miR-124-3p in GC tissues, thereby promoting integrin-β3 (ITGB3) expression and the proliferation, migration, and invasion of GC cells [123]. LncRNA-SND1-IT1 has a positive effect on transforming growth factor-β1 (TGF-β1) in promoting the occurrence of EMT in gastric cancer, and lncRNA SND1-IT1 can specifically bind to miR-124 to further promote the occurrence of EMT in gastric cancer [122].

We summarize recent studies on the function of lncRNAs in gastric cancer in Table 3.

### PIWI-Interacting RNAs in gastric cancer

PIWI-interacting RNA (piRNA) is a small molecule ncRNA that can interact with PIWI proteins, first identified in the developing Drosophila melanogaster, usually between 24–30 nt in length, and lacks sequence conservation between organisms [119]. The human stomach contains 8759 piRNAs, with 50 of these showing significant differential expression in tumor tissues. This suggests that piRNAs hold promise as potential cancer biomarkers for further research. Additionally, other studies have indicated that the expression patterns of piRNAs can effectively distinguish between cancerous and healthy tissues [161]. Although there have been many studies on piRNA in the last decade, few have studied the role of piRNA in gastric carcinogenesis.

PiR-823 showed a tumor suppressive impact, as evidenced by the considerable down-regulation of piR-823 expression in gastric cancer tissues and the inhibition of cell proliferation following an increase in piR-823 expression in gastric cancer cells. The study found that the level of piR823 was positively correlated with T stage and distant metastasis [162,163]. Unlike piR-823, piR-651 is overexpressed in GC and is associated with the TNM stage. Knockdown of piR-651 inhibited the growth of gastric cancer cells and caused them to stop growing in the G2/M stage, suggesting that piR-651 plays a role in promoting cancer in the development of gastric cancer [164]. The study showed that stomach cancer patients had lower peripheral blood levels of piR-651 and piR-823 compared to healthy people. In addition, piR-651 and piR-823 have higher sensitivity and specificity compared to the positive detection rates of CEA and CA19-9 [163]. The study found that the expression of piR-019308, piR-004918, and piR-018569 in the serum exosomes of gastric cancer patients was significantly higher than that of normal people, and the expression levels of piR-004918 and piR-019308 were related to metastasis [165]. Research indicates that PIWIL1 is significantly expressed in tissues and cell lines of gastric cancer. Inhibiting the expression of PIWIL1 can reduce the proliferation, migration, tumorigenesis, and metastasis of cancer cells. PIWIL1 has been identified as a potential target for the precision treatment of gastric cancer [166]. Individuals suffering from gastric cancer exhibit notable amounts of PiR-1245 in their gastric juices. As per research findings, those gastric cancer patients who show the presence of piR-1245 in their gastric juices have a poorer overall survival rate as well as a lower progression-free survival rate. Moreover, PiR-1245 is strongly associated with both tumor size and the TNM stage [167].

Since the function of piRNAs in somatic cells has not been determined, the possible regulatory role of piRNAs in non-cancerous gastric tissues and gastric cancer tissues remains difficult to determine. PiRNA shows great potential as a molecular marker for both diagnosing and treating gastric cancer. Although investigations into piRNA’s role in this context are still emerging, the notably elevated levels of piRNA found in gastric tissues indicate it may have a significant function in the pathophysiology of gastric cancer.

We summarize recent studies on the function of piRNAs in gastric cancer in Table 3.

## Conclusion

Even though the cure rate for early gastric cancer is extremely high, the majority of patients are diagnosed at the intermediate and advanced stages of the disease, with varying degrees of distant metastasis. This is due to the disease’s atypical clinical symptoms in its early stages, along with its highly invasive and metastatic capabilities. The challenges in diagnosing early gastric cancer and treating advanced gastric cancer underscore the necessity to explore more precise and targeted techniques for early diagnosis and develop more effective medications for intermediate and advanced cases.

While a growing number of biomarkers and immune checkpoints have been identified and therapeutics have been developed for them, only a small percentage of patients may benefit from them. For example, EBV(+)GC is more sensitive to the immune response, which may be because EBV(+)GC can produce a strong inflammatory response from neoantigens, resulting in the invasion of a large number of lymphocytes. Most other gastric cancers, on the contrary, are immunologically less sensitive. Studies have shown that patients with dMMR, high TMB, EBV+, and PD-L1 positivity are more likely to respond better to PD-L1 inhibitors [168], but there is still a lack of drugs that can treat most patients with cancer, and immune checkpoint inhibitors have shown efficacy in only about 10% of patients with gastric cancer [105].

In the conventional diagnosis of gastric cancer, endoscopy boasts the benefits of providing an intuitive and accurate diagnosis. However, it is an invasive procedure, and there’s a chance of overlooking the diagnosis of minor lesions. Imaging examination can provide a comprehensive evaluation of the tumor and has staging value, but it has low sensitivity for the diagnosis of early gastric cancer and has a radiation risk. Histopathological analysis can provide the most accurate pathological diagnosis, but the acquisition of tissue samples relies on invasive examination, and secondly, there may be sampling errors, which cannot fully represent tumor heterogeneity. Serum markers can be used for dynamic monitoring and diagnostic aids, but their sensitivity and specificity are low, and they cannot be used for diagnosis alone. Comparatively, some ncRNAs have begun to be used as new targets for the treatment of gastric cancer. Some ncRNAs are abundant in the serum or urine of gastric cancer patients and serve as potential diagnostic markers or prognostic indicators. NcRNAs in blood can be used as a non-invasive diagnostic tool, and liquid biopsy technology can be used to achieve early diagnosis and dynamic monitoring of gastric cancer. A serum diagnostic marker based on m6A-targeted mirna performed well in differentiating GC, and its diagnostic performance was not affected by sex, age, or benign disease. The area under the curve of m6A level in peripheral blood RNA combined with CEA and CA199 for the diagnosis of GC was larger than that of m6A alone [169]. The accuracy and specificity of a single ncRNA as a diagnostic marker are limited, and the combined detection of multiple ncRNAs in the future can improve the diagnostic efficacy. In addition, there are differences in ncRNA expression profiles in patients with different subtypes of gastric cancer, and an in-depth study of the relationship between ncRNA expression characteristics and molecular typing and clinicopathological characteristics of gastric cancer can achieve disease stratification. Personalized diagnostic strategies based on the ncRNA expression profile of individual patients can help identify high-risk groups and guide subsequent precision treatment.

Traditional treatment methods mainly include surgery, radiotherapy, chemotherapy, and targeted therapy, which are mature and widely used but have large side effects and insufficient early diagnosis. The treatment methods of ncRNA mainly include the regulation of ncRNA expression, gene therapy, etc., which have the advantages of precision treatment, reducing side effects, and reversing drug resistance. However, ncRNA technology is not yet fully mature, and most of the existing ncRNA-based targeted therapies are in the experimental stage. The treatment regimen needs to be optimized in the future. On the one hand, it is necessary to improve the efficiency of ncRNA delivery and develop safer and more efficient vector systems to ensure the precise delivery of therapeutic ncRNAs to tumor cells. On the other hand, in-depth research on the mechanism of action of ncRNA, design more specific targeted drugs, reduce off-target effects, and improve the safety and efficacy of treatment. Emerging technologies and new methods will bring better results, and we can seek to combine ncRNA-targeted methods with immunotherapy or other treatments. The combination of ncRNA therapy with traditional chemotherapy, radiotherapy, and immunotherapy is an important development direction. ncRNA can regulate the sensitivity of tumor cells to chemoradiotherapy and enhance the therapeutic effect. For example, by modulating specific ncRNAs to overcome drug resistance in gastric cancer cells, chemotherapy drugs work better. At the same time, ncRNA combined with immunotherapy can regulate the tumor immune microenvironment and enhance the body’s anti-tumor immune response. Dynamic monitoring of ncRNA expression changes during treatment can reflect the treatment effect in real time and guide the adjustment of the treatment plan. By establishing a correlation model between ncRNA expression and treatment response and prognosis, the accuracy of treatment monitoring and prognosis prediction was realized. Although non-coding RNAs have shown great potential in the diagnosis and treatment of gastric cancer, there are still some challenges. The stability of non-coding RNAs, the standardization of detection methods, and the specificity and safety of targeted therapies still need to be further studied. In addition, how to better integrate non-coding RNA markers with existing diagnostic and therapeutic processes is also a direction that needs to be explored in the future. In general, non-coding RNA has broad application prospects in the diagnosis and treatment of gastric cancer, and with the continuous progress of technology and in-depth research, it is expected to bring more accurate and effective diagnosis and treatment options for gastric cancer patients.

The field of gastric cancer should, in conclusion, focus on improving early-stage cancer detection, monitoring gastric precancers more appropriately, accurately identifying patients who may benefit from particular treatments, and better understanding the incidence of gastric cancer in order to find new therapeutic targets for advanced GC.

## Data Availability

Not applicable.
